# NF-κB1 Haploinsufficiency Causing Immunodeficiency and EBV-Driven Lymphoproliferation

**DOI:** 10.1007/s10875-016-0306-1

**Published:** 2016-06-23

**Authors:** Heidrun Boztug, Tatjana Hirschmugl, Wolfgang Holter, Karoly Lakatos, Leo Kager, Doris Trapin, Winfried Pickl, Elisabeth Förster-Waldl, Kaan Boztug

**Affiliations:** St. Anna Kinderspital, Department of Pediatrics, Medical University of Vienna, Vienna, Austria; CeMM Research Center for Molecular Medicine of the Austrian Academy of Sciences, Vienna, Lazarettgasse 14 AKH BT 25.3, Vienna, Austria; Institute of Immunology, Center for Pathophysiology, Infectiology, and Immunology, Medical University of Vienna, Vienna, Austria; Department of Pediatrics and Adolescent Medicine, Medical University Vienna, Vienna, Austria; Ludwig Boltzmann Institute for Rare and Undiagnosed Diseases, Lazarettgasse 14 AKH BT 25.3, Vienna, Austria

**Keywords:** Combined immunodeficiency, EBV lymphoproliferative disease, NF-κB1, haploinsufficiency

## Abstract

**Purpose:**

NF-κB signaling is critically important for regulation of both innate and adaptive immune responses. While activation of NF-κB has been implicated in malignancies such as leukemia and lymphoma, loss-of-function mutations affecting different NF-κB pathway components have been shown to cause primary immunodeficiency disorders. Recently, haploinsufficiency of NF-κB1 has been described in three families with common variable immunodeficiency (CVID).

**Methods and Results:**

We studied a patient with recurrent respiratory infections and bacterial parapharyngeal abscess. Immunological investigations revealed normal total B- cell numbers, but hypogammaglobulinemia, decreased frequencies of class-switched B cells and impaired T-cell proliferation. Targeted next-generation sequencing using a custom-designed panel comprising all known PID genes (IUIS 2014 classification) and novel candidate genes identified a novel heterozygous frameshift mutation in the *NFKB1* gene leading to a premature stop codon (c.491delG; p.G165A*31). We could show that the mutation leads to reduced phosphorylation of p105 upon stimulation, resulting in decreased protein levels of p50. The further disease course was mainly characterized by two episodes of severe EBV-associated lymphoproliferative disease responsive to rituximab treatment. Due to disease severity, the patient is considered for allogeneic hematopoietic stem cell transplantation. Interestingly, the father carries the same heterozygous *NFKB1* mutation and also shows decreased frequencies of memory B cells but has a much milder clinical phenotype, in line with a considerable phenotypic disease heterogeneity.

**Conclusions:**

Deficiency of NF-κB1 leads to immunodeficiency with a wider phenotypic spectrum of disease manifestation than previously appreciated, including EBV lymphoproliferative diseases as a hitherto unrecognized feature of the disease.

**Electronic supplementary material:**

The online version of this article (doi:10.1007/s10875-016-0306-1) contains supplementary material, which is available to authorized users.

## Introduction

NF-κB signaling is a complex, conserved pathway critical for regulation of both innate and adaptive immune reactions in response to various stimuli. Central players in this pathway in mammals comprise five transcription factors, including REL-A/p65, REL-B, c-REL, NF-κB1/p50, and NF-κB2/p52 [1]. These transcription factors share a Rel homology domain (RHD) in their N-terminal DNA-binding domain responsible for formation of homo- and heterodimers which can bind to DNA to regulate target gene expression [1].

NF-κB signaling cascades are differentiated into two major subsets termed “canonical” and “non-canonical” pathways. Canonical NF-κB signaling may be activated upon contact with numerous inflammatory stimuli including pattern recognition receptors and cytokine receptors and signals through a complex consisting of IKKα/IKKβ/IKKγ and the formation of p50/p65 heterodimers (reviewed in [2]). By contrast, non-canonical NF-κB signaling is activated through tumor necrosis family receptors upon binding of BAFF, lymphotoxin β, or CD40 ligand, and is predominantly known to regulate immune cell differentiation and also formation of secondary lymphoid organs (reviewed in [3]). Notably, recent studies have demonstrated considerable cross talk between both arms of NF-κB signaling; thus, these signaling cascades are highly interconnected [4].

In view of the central role of NF-κB signaling for multiple processes in both innate and adaptive immunity, it is not surprising that aberrant NF-κB signaling may be implicated in human disease pathogenesis. Thus, while excessive activation of NF-κB has been shown to play a role in hematological malignancies [5], loss-of-function mutations in genes of the canonical and non-canonical NF-κB pathway have been associated with distinct types of primary immunodeficiency disorders including deficiencies of NEMO [6] or IKKβ [7] and BAFF receptor [8], CD40/CD40 ligand [9, 10], NIK [11], AID [12], or autosomal dominant mutations affecting NFκB2 [13, 14], respectively.

Very recently, haploinsufficiency of NF-κB1 has been identified as a novel genetic etiology of a subtype of common variable immunodeficiency (OMIM616576) [15]. The gene encodes for the p105 protein which is processed to the “active” p50 subunit implicated in the canonical NF-κB pathway. As p50 lacks a C-terminal transcription activation domain, p50 can only activate transcription by formation of heterodimers, for example with REL-A, while p50 homodimers act as transcriptional repressors [16].

Here, we identify a novel mutation of *NFKB1* in a patient with combined immunodeficiency with impaired B and T cell functions and presentation with severe Epstein-Barr virus (EBV)-associated lymphoproliferation as a hitherto unrecognized clinical disease manifestation.

## Methods

### Patients

All patient material was obtained in accordance with the Declaration of Helsinki. The study was approved by the ethics committee of the Medical University of Vienna.

### DNA Isolation and Preparation

Genomic DNA (gDNA) was isolated from EDTA blood using an adapted protocol of the Wizard^®^ Genomic DNA Purification Kit (Promega). gDNA isolation from buccal swabs was performed using the QIAamp^®^ DNA Mini Kit (Qiagen), following the spin protocol of the QIAamp^®^ DNA Mini and Blood Mini Handbook.

For library preparation, gDNA was diluted and then measured on a Qubit 2.0 Fluorometer (Invitrogen/Life Technologies) for a total concentration of 200 ng.

### Targeted Exome Sequencing

The patient sample was screened for disease-causing variants by a custom-designed targeted enrichment approach (HaloPlex^™^/Agilent Technologies) followed by next-generation sequencing on a HiSeq3000 (Illumina) platform as described previously [17]. In brief, enrichment of the targeted plus 25-bp flanking region was accomplished using the HaloPlex Target Enrichment System (Agilent Technologies Inc., 2013), based on a molecular inversion probe strategy. Library preparation was performed according to the manufacturer’s instruction. In brief, 200 ng of gDNA was digested by eight pairs of restriction enzymes, followed by bar code indexing and hybridization to custom-designed capture probes for 16 h at 54 °C. Thereafter, the circularized biotinylated target-probe complexes were extracted using magnetic streptavidin beads. The final steps included nick ligation, PCR library amplification, and AmPure XP bead (Beckman Coulter, Inc.) purification prior to qualitative and quantitative assessment of the DNA library using a 2100 Bioanalyzer instrument (Agilent). Next-generation sequencing was performed in a 150-bp paired-end mode using a HiSeq3000 (Illumina) platform.

### Data Analysis

The gross data analysis pipeline included adapter trimming of Illumina sequences (Trimmomatic), Burrows-Wheeler Aligner (BWA) for sequence alignment to the human genome 19 (hg19), Indel Realignment on both sequence aliquot and sample level via Genome Analysis Toolkit (GATK; Broad Institute), Base Quality Score Recalibration (GATK), Haplotype Calling (GATK), and Variant Annotation (SnpEFF, GATK). Thereafter, variant filtering included the criteria of being rare (MAF ≤ 0.01), non-synonymous, and within the coding region of the targeted genes. In addition to published data, we assessed the potential relevance of variants by recurrence within ExAC browser (Exome Aggregation Consortium Cambridge) and in our internal dataset comprising of more than 300 sequenced individuals to date. Of note, variants with a VQSLOD score (the log odds of being a true variant versus being false) below 99.9 % of the truth set of a trained Gaussian mixture model can be considered as false positives and are thus not shown herein.

### Coverage

The GATK CallableLoci tool was executed in order to assess the proportion of callable bases, as determined by sequencing depth and mapping quality per interrogated position. Hence, targeted genomic regions were assigned different quality categories (pass, no coverage, low coverage, excessive coverage, poor mapping quality) and summarized in a BED file. According to this analysis, 99.76 % of enriched exonic bases were considered callable with a minimum read depth of 4 and minimum mapping quality score of 10.

### Variant Validations

Sanger validation was performed according to standard capillary sequencing protocol using a 3130xl Genetic Analyzer instrument.

### Flow Cytometry-Based Immunophenotyping and T Cell Proliferation Assays

These analyses were carried out as described previously [18].

### Western Blot Analysis

Following 3 h of starvation in IMDM without FCS, EBV-transformed B lymphocytes derived from the affected individual and the father, both harboring the heterozygous *NFKB1* c.491delG mutation, as well as from a healthy donor individual, were stimulated with 50 ng/ml PMA plus 1 μg/ml ionomycin (both from Sigma Aldrich) for 30 and 60 min, as indicated. Cells were lysed on ice (lysis buffer 20 mM Tris–HCl, pH 7.5, 150 mM NaCl, 2 mM EDTA, 1 μM Na_3_VO_4_, 50 mM NaF, 1 % Triton X-100; Protease Inhibitor Cocktail) and total protein concentrations determined via Bradford (Biorad). Fifty micrograms per sample were loaded and protein size fractionated using a 12 % polyacrylamide gel. Proteins were transferred onto a PVDF (GE Healthcare) membrane (0.45-μm pore size) overnight at 4 °C (200 mA) in accordance with standard Western blotting protocols. P105 and p50 proteins were detected using a rabbit antibody raised against the NF-κB1 N-terminus (no. 3035, Cell Signaling, New England Biolabs); phosphorylated p105 (Ser933) was detected using a monoclonal rabbit antibody (no. 4806, Cell Signaling). GAPDH served as loading control and was detected using a mouse monoclonal antibody (sc-365062; Santa Cruz). Anti-rabbit secondary antibody (Biorad) and anti-mouse secondary antibody (BD Biosciences) coupled to horseradish-peroxidase were used for signal detection. Immunoreactive bands were visualized with Amersham ECL Select Western Blotting Substrate (GE Healthcare).

## Results

The index patient was born to non-consanguineous parents of Caucasian origin. Following an unremarkable first year of life, she started to have recurrent mainly upper respiratory tract infections in early childhood. At 5 years of age, tonsillectomy and adenotomy were performed. In the following years, the patient suffered from recurrent upper airway infections which were mostly self-limiting.

**Fig. 1:**
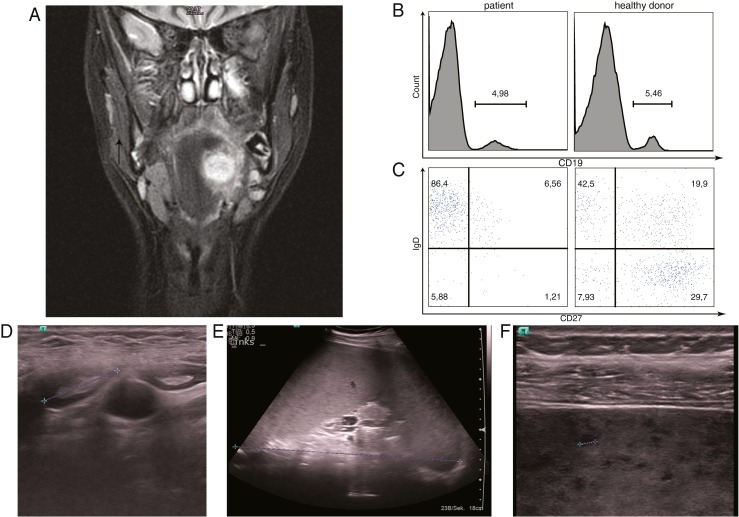
Clinical and immunological phenotype. Initially, the index patient presented with a parapharyngeal abscess which was surgically drained (**a**). Immunological assessment revealed normal total number of B cells (**b**) but decreased numbers of non-switched (CD27^+^IgD^+^) and switched (CD27^+^IgD^−^) memory B cells in comparison to healthy donor. **c** At the age of 18 years, the patient showed EBV lymphoproliferative diseases including EBV reactivation, cervical lymphadenopathy (**d**), splenomegaly (**e**), and multiple splenic lesions (**f**), all of which normalized upon treatment with anti-CD20 (rituximab)

At the age of 15 years, she was referred to our hospital for the first time with fever and problems with swallowing and was found to have parapharyngeal abscess (Fig. 1a) and cervical lymphadenopathy. Blood results showed leukopenia (1.89 G/l), neutropenia (0.06 G/l), thrombocytopenia (minimum 119 G/l), and elevated C-reactive protein levels (CRP; 174 mg/l). No hepatosplenomegaly was apparent. The parapharyngeal abscess was surgically drained; histological evaluation revealed chronic abscess-forming inflammation, microbiologic culture infection with viridans group streptococci and actinomyces. Decreased leukocyte and thrombocyte counts prompted bone marrow aspiration, which revealed predominantly immature granulopoesis, compatible with bacterial infection, but no evidence of myelodysplasia or malignant disease. The patient improved with antibiotic therapy and absolute neutrophil counts normalized concomitantly. Immunological investigations following recovery revealed low absolute numbers of CD19^+^ B cells with reduced non-switched and switched memory cells (IgD^+^/CD27^+^; IgD^−^/CD27^+^) and low immunoglobulin levels (Fig. 1b, c and Table 1). Absolute numbers of T cells were normal; however, proliferation assays revealed impaired T-cell function in response to tetanus toxoid, CD3, and PMA with normal response to PHA (Table 1). The patient was started on intravenous and subsequently subcutaneous immunoglobulin substitution. Absolute neutrophil counts and thrombocytes were repeatedly decreased (thrombocytes with a minimum of 32 G/l) with spontaneous normalization; however, anti-granulocyte and anti-thrombocyte antibodies were negative. Few months later, she developed fever, neutropenia (0.1 G/l), elevated CRP (80 mg/l), esophagitis, cervical, axillary and supraclavicular lymphadenopathy, and mild hepatosplenomegaly. Axillary lymph node biopsy showed reactive changes and the patient improved with antibiotic therapy.

**Table 1:** Laboratory findings

Lymphocyte subsets								Patient’s father
Test date	04/2012	10/2012	11/2013	02/2015	04/2015	05/2015	10/2015	02/2016
CD3 absolute ×10E9/l (0.8–3.5)	1	1.2	1.28	0.94	0.84	0.83	0.84	1.72
CD4 absolute ×10E9/l (0.4–2.1)	0.42	0.5	0.61	0.66	0.45	0.39	0.55	0.92
CD8 absolute ×10E9/l (0.2–1.2)	0.49	0.5	0.55	0.23	0.34	0.38	0.25	0.8
CD4/8 (0.9–3.4)	0.9	1	1.11	2.91	1.34	1.03	2.19	1.15
DNT (a/b^+^/CD4^−^/CD8^−^)	ND	2	ND	5	ND	2	2	ND
CD45RA %	54	52	43	18	27	ND	ND	39
CD45R0^+^ memory %	23	26	40	77	67	ND	ND	36
CD19 absolute ×10E9/l (0.2–0.6)	*0.08*	*0.06*	*0.05*	*0.02*	*0* ^a^	*0* ^a^	*0.01* ^a^	*0.89*
IgD^+^/CD27^+^ % CD19^+^ (6.1–16.9)	*3.5*	ND	7.7	ND	ND	ND	ND	*49.9*
IgD^−^/CD27^+^ % CD19^+^ (4.1–18.7)	*0.9*	ND	*3.8*	ND	ND	ND	ND	*1.05*
CD56/CD3^−^ absolute ×10E9/l (0.07–1.2)	0.15	0.1	0.17	*0.04*	0.09	0.09	0.1	0.28
Blood counts								
Test date	01/2012	02/2012	08/2012	11/2013	02/2015	04/2015	10/2015	02/2016
WBC (G/l) (4–12)	*1.11*	*3.7*	*2.3*	*2.5*	*2.3*	4	*2.1*	9.9
ALC (G/l) (0.8–3.5)	*0.7*	1.08	1.18	1.58	1.04	0.96	0.97	3.07
ANC (G/l) (1.9–8.00)	*0.1*	1.98	*0.68*	*0.3*	*0.97*	2.8	*0.99*	6.24
Thrombocytes (G/l) (140–400)	*119*	193	*97*	147	*115*	225	*70*	237
T cell proliferation								
Test date	04/2012				03/2014			
Stimulus	PMA	PHA	CD3 Ab	Tetanus toxoid	PMA	PHA	CD3 Ab	Tetanus toxoid
Patient (×10E3 cpm)	*9*	88	*68*	*4*	15	83	*51*	*2*
Healthy control (×10E3 cpm)	34	66	146	29	22	108	72	31
	12/2015							
Patient’s father (×10E3 cpm)	259	288	184	*23*				
Healthy control (×10E3 cpm)	53	156	93	75				
Vaccination titers	Tetanus	Diphteria	*Haemophilus influenzae* B	Pneumococcus				
Patient (test date 04/2012)	0.5 IU/ml	*0.04 IU/ml*	0.34 μg/l	33.88 mg/l				
Patient’s father (test date 02/2016)	1.7 IU/ml	*0.04 IU/ml*	2.04 μg/l	ND				
	IgG	IgG1	IgG2	IgG3	IgG4	IgM	IgA	
Immunoglobulins (mg/dl)	(700–1600)	(280–800)	(115–570)	(24–125)	(5.2–125)	(40–230)	(70–400)	
Patient (test date 03/2012)	*232*	*148*	*61*	*22*	*0.3*	*1.2*	*0.05*	
Patient’s father (test date 02/2016)	*409*	*165*	204	*18*	20	41	*23*	

Values in brackets show reference ranges. Abnormal values are printed in italic

*WBC* white blood cells, *ALC* absolute lymphocyte count, *ANC* absolute neutrophil count, *PMA* phorbol-12-myristate-13-acetate, *PHA* phytohemagglutinin, *Ab* antibody

^a^Post four cycles of rituximab

**Fig. 2:**
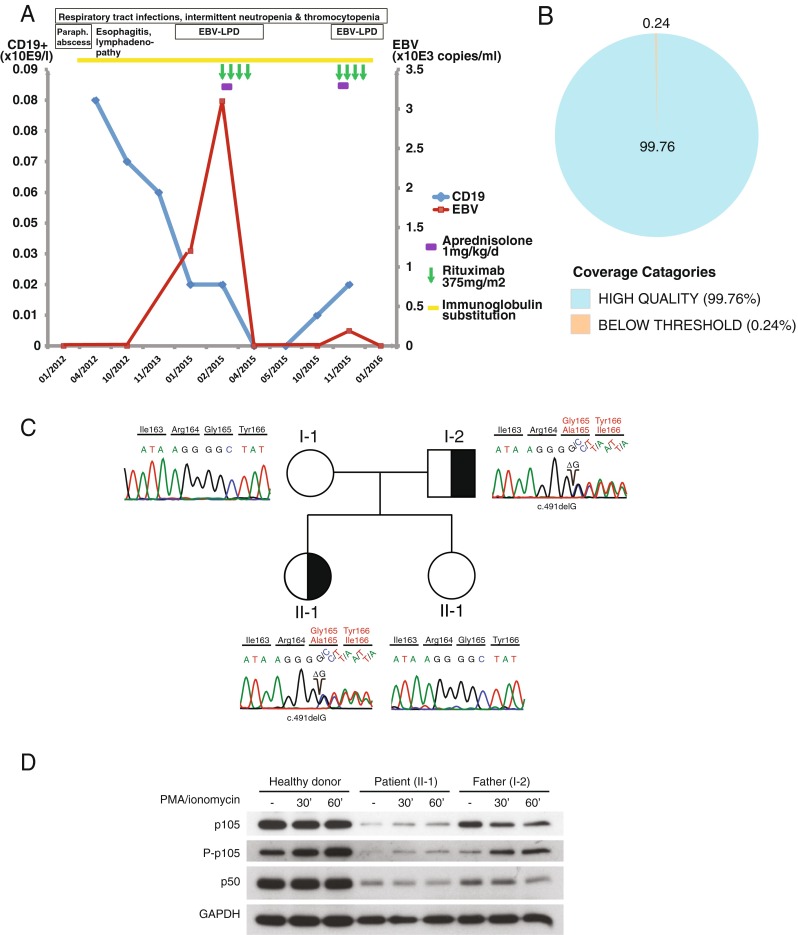
Identification of a disease-causing mutation in. Disease severity and complications increased over time with two severe episodes of EBV-associated lymphoproliferation within one year (**a**). The patient was assessed using a targeted, next-generation sequencing-based gene panel with high on-target coverage (**b**). A heterozygous mutation in the RHD domain of the *NFKB1* gene was identified, leading to a frameshift and a subsequent stop codon (c.491delG; p.G165A*31). The patient’s father was found to be a carrier of the disease (**c**) and shows an aberrant B cell immunophenotype despite his mild clinical manifestation (Table 1). The mutation leads to reduced phosphorylation of p105 upon stimulation in both index patient (II-1) and father (I-2), resulting in decreased protein levels of p50 (**d**)

In the following years, the patient had three to four uncomplicated infections per year (sinusitis, pharyngitis, urinary tract infection). She complained of intermittent arthralgia; autoantibodies were repeatedly negative (rheumatoid factor (RF), anti-neutrophil cytoplasmatic antibody (ANCA), anti-cardiolipin antibodies, anti-nuclear antibody (ANA)). At the age of 18 years, splenomegaly (21 cm) with multiple hypodense changes, hepatomegaly (20 cm), and generalized lymphadenopathy occurred (not shown). Blood results showed reactivation of Epstein-Barr-virus (EBV) with 1.2 × 10E3 copies/ml. Within a short time, the patient deteriorated with high fever, elevated CRP (50 mg/l), and increasing hepatosplenomegaly and lymphadenopathy (liver 22 cm, spleen 26 cm; Fig. 1d). Further investigations were not suspicious for macrophage-activating syndrome. With rising EBV load (maximum 3.1 × 10E3 copies/ml), EBV-associated lymphoproliferative disease (EBV-LPD) was suspected. The patient was started on corticosteroid treatment (1 mg/kg) and weekly rituximab (375 mg/m^2^) was administered four times. Prompt clinical improvement was noted, EBV load turned negative, and CD19^+^ B cells were not detectable for 8 months (Fig. 2a). However with reemergence of peripheral blood B cells, a similar clinical picture with fever, lymphadenopathy, and hepatoslenomegaly reoccured and reactivation of EBV (maximal load 1.2 × 10E2 copies/ml) was detected. The patient was started on cortisone and four courses rituximab, which has led to remission of the disease again. In view of the relatively severe disease course, HLA typing of the patient and family has been initiated to prepare for allogeneic hematopoietic stem cell transplantation. On inquiry of the patient’s family, the patient’s father declared he had been suffering from frequent non-severe respiratory tract infections but not requiring regular medical attention.

### Identification of Underlying Genetic Defect in *NFKB1*

Given the unclear origin of immunodeficiency, we assessed the patient using our custom-designed next-generation sequencing (NGS)-based panel which covers 419 genes, including all known primary immunodeficiency disease (PID)-causing genes according to the IUIS classification from 2014 [19] and several PID candidate genes in which defects are likely to be causative for monogenic primary immunodeficiencies. Our sequencing approach yielded very high on-target coverage (Fig. 2b). The analysis of this patient did not reveal any homozygous variant. Among few other heterozygous variants (Supplementary Table 1), we could, however, identify a novel frameshift mutation in *NFKB1* (ENST00000226574 c.491delG; p.G165A*31), which resides within the N-terminal RHD and results in a premature stop codon. While the mother and sister were wild type at this position, genetic analysis revealed the same *NFKB1* mutation in the patient’s father as in our index patient (Fig. 2c), however, associated with a mild clinical phenotype. While his absolute numbers of CD19^+^ B cells were mildly elevated (0.89 × 10E9/l), switched memory B cells (IgD^+^/CD27^+^ 49.9 %; IgD^−^/CD27^+^ 1.05 %) as well as IgG (including subclasses IgG1 and IgG3) and IgA were decreased. T cell proliferation assays revealed decreased response to T cell recall antigens such as tetanus toxoid, however not as pronounced as in the index patient, while T cell responses to stimulation with PHA, PMA, or anti-CD3 were normal. Despite a previous immunization, vaccination titers to diphtheria toxin were low, comparable with the index patient’s results (Table 1).

Western blot analysis revealed detectable but severely decreased levels of p50 in both the index patient (II-1) and her father (I-2). Upon stimulation with PMA and ionomycin, we observed that phosphorylation of p105 was markedly lower than in a healthy control in line with a signaling defected conferred by the *NFKB1* mutation detected in both the index patient and her father (Fig. 2d). No truncated p50 protein was detected (Supplementary Figure 1).

## Discussion

Identification of the genetic etiologies underlying primary immunodeficiencies has contributed considerably to our understanding of core signaling processes in the human immune system. In particular, the advent of NGS technologies has accelerated disease gene discovery for rare monogenetic disorders altogether [20]. While historically large pedigrees were necessary to identify novel causative gene defects and the majority of identified defects were inherited in an autosomal recessive manner (in part because studies in consanguineous pedigrees were used to identify underlying gene defects), more recently, an increasing number of autosomal dominant (AD) PIDs has been discovered, including such that work through AD gain-of-function mutations (e.g., activated PIK3δ syndrome [21–24]) or haploinsufficiency (e.g., CTLA4 haploinsufficiency syndrome [25, 26]).

Recently, haploinsufficiency of the NF-κB subunit p50 has been described in three families [15]. Mutations were located within the RHD domain and lead to altered or absent p50 protein. Clinically, the patients mainly presented with recurrent respiratory tract infections, lymphadenopathy, and chronic-obstructive pulmonary disease, with marked disease severity. Laboratory findings included hypogammaglobulinemia and abnormal response to immunizations in some patients but normal numbers and function of T cells at least in some patients, in line with a diagnosis of common variable immunodeficiency [15].

Here, we describe a young adult patient with a novel frameshift mutation in *NFKB1*, also residing within the RHD. Clinically, our patient initially had a fairly mild disease course during childhood but then increasingly suffered from upper respiratory tract infections. The immunological investigations, prompted by diagnosis of a parapharyngeal abscess with uncommon microbes, demonstrated hypogammaglobulinemia with paucity of class-switched B cells and impaired generation of antibodies upon vaccination, but also impaired T-cell proliferative responses, suggestive of a picture of combined immunodeficiency. The functional impairment of T cells likely underlies the susceptibility to EBV-induced lymphoproliferation, which is a hitherto unrecognized feature of NF-κB1 haploinsufficiency. Several immunodeficiency disorders associated with EBV lymphoproliferation have been described, including deficiency of ITK, XIAP, STK4, SH2D1A, and CD27 [27–30]. The coding sequences of these genes were fully covered, and no mutations were detected in our targeted NGS panel (Supplementary Table 2). A defective function of T and NK cells is thought to lead to immune dysregulation and lymphoproliferation [30]. While absolute numbers of T cells were normal in our patient, T-cell proliferation assays showed markedly decreased results possibly contributing to EBV lymphoproliferation. Haploinsufficiency of NF-κB1 has been described so far as a predominant defect of B cells and has been assigned to the group of CVID. In view of the diminished function of T cells in our patient and the severe EBV lymphoproliferative disease, we suggest that haploinsufficiency of NF-κB1 may cause a phenotype of combined immunodeficiency at least in some patients.

Autosomal dominant inherited immunodeficiencies are often associated with incomplete disease penetrance and heterogeneity of clinical and laboratory manifestations. It seems that this also applies to haploinsufficiency of NF-κB1 as our patient’s father carries the same *NFKB1* mutation as the index patient, however associated with milder clinical and laboratory findings. In view of the disease heterogeneity, therapeutic implications cannot be uniform and need to be adapted to the presentation of the individual patient. In our index patient, disease severity and complications increased over time with two severe episodes of EBV-associated lymphoproliferation within one year. Hence, we are currently considering the patient for allogeneic hematopoietic stem cell transplantation. Comprehensive studies with larger numbers of *NFKB1* mutant patients are urgently needed to exhibit the full spectrum of clinical disease manifestations of NF-κB1 haploinsufficiency, identify potential genotype-phenotype correlations, and rationally define criteria for treatment in specific patients and circumstances.

## Conclusions

NF-κB1 haploinsufficiency is a primary immunodeficiency resulting in impaired function of B cells and can, at least in some patients, lead to decreased function of T cells as well. The clinical phenotype of NF-κB1 haploinsufficiency may be more complex with a more heterogeneous clinical disease pattern than previously described, including EBV-associated lymphoproliferation as a previously unrecognized clinical feature of the disease. Future studies will need to delineate the full clinical and immunological disease spectrum and prospectively assess different therapeutic approaches.

## Electronic Supplementary Material

Below is the link to the electronic supplementary material.

ESM 1 (PDF 4973 kb)

ESM 2 (DOCX 18 kb)

ESM 3 (DOCX 31 kb)

## References

[1] Hayden MS, Ghosh S (2011). NF-kappaB in immunobiology. Cell Res.

[2] Vallabhapurapu S, Karin M (2009). Regulation and function of NF-kappaB transcription factors in the immune system. Annu Rev Immunol.

[3] Shih VF, Tsui R, Caldwell A, Hoffmann A (2011). A single NFkappaB system for both canonical and non-canonical signaling. Cell Res.

[4] Oeckinghaus A, Hayden MS, Ghosh S (2011). Crosstalk in NF-kappaB signaling pathways. Nat Immunol.

[5] Gasparini C, Celeghini C, Monasta L, Zauli G (2014). NF-kappaB pathways in hematological malignancies. Cell Mol Life Sci.

[6] Doffinger R, Smahi A, Bessia C, Geissmann F, Feinberg J, Durandy A (2001). X-linked anhidrotic ectodermal dysplasia with immunodeficiency is caused by impaired NF-kappaB signaling. Nat Genet.

[7] Pannicke U, Baumann B, Fuchs S, Henneke P, Rensing-Ehl A, Rizzi M (2013). Deficiency of innate and acquired immunity caused by an IKBKB mutation. N Engl J Med.

[8] Warnatz K, Salzer U, Rizzi M, Fischer B, Gutenberger S, Bohm J (2009). B-cell activating factor receptor deficiency is associated with an adult-onset antibody deficiency syndrome in humans. Proc Natl Acad Sci U S A.

[9] Allen RC, Armitage RJ, Conley ME, Rosenblatt H, Jenkins NA, Copeland NG (1993). CD40 ligand gene defects responsible for X-linked hyper-IgM syndrome. Science.

[10] Ferrari S, Giliani S, Insalaco A, Al-Ghonaium A, Soresina AR, Loubser M (2001). Mutations of CD40 gene cause an autosomal recessive form of immunodeficiency with hyper IgM. Proc Natl Acad Sci U S A.

[11] Willmann KL, Klaver S, Dogu F, Santos-Valente E, Garncarz W, Bilic I (2014). Biallelic loss-of-function mutation in NIK causes a primary immunodeficiency with multifaceted aberrant lymphoid immunity. Nat Commun.

[12] Revy P, Muto T, Levy Y, Geissmann F, Plebani A, Sanal O (2000). Activation-induced cytidine deaminase (AID) deficiency causes the autosomal recessive form of the hyper-IgM syndrome (HIGM2). Cell.

[13] Chen K, Coonrod EM, Kumanovics A, Franks ZF, Durtschi JD, Margraf RL (2013). Germline mutations in NFkB2 implicate the noncanonical NF-kappaB pathway in the pathogenesis of common variable immunodeficiency. Am J Hum Genet.

[14] Lee CE, Fulcher DA, Whittle B, Chand R, Fewings N, Field M (2014). Autosomal-dominant B-cell deficiency with alopecia due to a mutation in NFkB2 that results in nonprocessable p100. Blood.

[15] Fliegauf M, Bryant VL, Frede N, Slade C, Woon ST, Lehnert K (2015). Haploinsufficiency of the NF-kappaB1 subunit p50 in common variable immunodeficiency. Am J Hum Genet.

[16] Pereira SG, Oakley F (2008). Nuclear factor-kappaB1: regulation and function. Int J Biochem Cell Biol.

[17] Erman B, Bilic I, Hirschmugl T, Salzer E, Cagdas D, Esenboga S (2015). Combined immunodeficiency with CD4 lymphopenia and sclerosing cholangitis caused by a novel loss-of-function mutation affecting IL21R. Haematologica.

[18] Salzer E, Kansu A, Sic H, Majek P, Ikinciogullari A, Dogu FE (2014). Early-onset inflammatory bowel disease and common variable immunodeficiency-like disease caused by IL-21 deficiency. J Allergy Clin Immunol.

[19] Al-Herz W, Bousfiha A, Casanova JL, Chatila T, Conley ME, Cunningham-Rundles C (2014). Primary immunodeficiency diseases: an update on the classification from the International Union of Immunological Societies Expert Committee for Primary Immunodeficiency. Front Immunol.

[20] Boycott KM, Dyment DA, Sawyer SL, Vanstone MR, Beaulieu CL (2014). Identification of genes for childhood heritable diseases. Annu Rev Med.

[21] Angulo I, Vadas O, Garcon F, Banham-Hall E, Plagnol V, Leahy TR (2013). Phosphoinositide 3-kinase delta gene mutation predisposes to respiratory infection and airway damage. Science.

[22] Deau MC, Heurtier L, Frange P, Suarez F, Bole-Feysot C, Nitschke P (2014). A human immunodeficiency caused by mutations in the PIK3R1 gene. J Clin Invest.

[23] Lucas CL, Kuehn HS, Zhao F, Niemela JE, Deenick EK, Palendira U (2014). Dominant-activating germline mutations in the gene encoding the PI(3)K catalytic subunit p110delta result in T cell senescence and human immunodeficiency. Nat Immunol.

[24] Lucas CL, Zhang Y, Venida A, Wang Y, Hughes J, McElwee J (2014). Heterozygous splice mutation in PIK3R1 causes human immunodeficiency with lymphoproliferation due to dominant activation of PI3K. J Exp Med.

[25] Schubert D, Bode C, Kenefeck R, Hou TZ, Wing JB, Kennedy A (2014). Autosomal dominant immune dysregulation syndrome in humans with CTLA4 mutations. Nat Med.

[26] Kuehn HS, Ouyang W, Lo B, Deenick EK, Niemela JE, Avery DT (2014). Immune dysregulation in human subjects with heterozygous germline mutations in CTLA4. Science.

[27] Speckmann C, Lehmberg K, Albert MH, Damgaard RB, Fritsch M, Gyrd-Hansen M (2013). X-linked inhibitor of apoptosis (XIAP) deficiency: the spectrum of presenting manifestations beyond hemophagocytic lymphohistiocytosis. Clin Immunol.

[28] Abdollahpour H, Appaswamy G, Kotlarz D, Diestelhorst J, Beier R, Schaffer AA (2012). The phenotype of human STK4 deficiency. Blood.

[29] Ghosh S, Bienemann K, Boztug K, Borkhardt A (2014). Interleukin-2-inducible T-cell kinase (ITK) deficiency—clinical and molecular aspects. J Clin Immunol.

[30] Salzer E, Daschkey S, Choo S, Gombert M, Santos-Valente E, Ginzel S (2013). Combined immunodeficiency with life-threatening EBV-associated lymphoproliferative disorder in patients lacking functional CD27. Haematologica.

