# Supplementary material for: NF-κB1 Haploinsufficiency Causing Immunodeficiency and EBV-Driven Lymphoproliferation
Source: J Clin Immunol. 2016 Jun 23;36:533–40. doi: 10.1007/s10875-016-0306-1 (PMC4940442; doi:10.1007/s10875-016-0306-1)
Supplement: Supplementary file 1 — (PDF 4973 kb) [file 10875_2016_306_MOESM1_ESM.pdf]

NFκB1 haploinsufficiency causing combined immunodeficiency and EBV-driven lymphoproliferation

Journal of Clinical Immunology

Heidrun Boztug, Tatjana Hirschmugl, Wolfgang Holter, Karoly Lakatos, Leo Kager, Doris Trapin, Winfried Pickl, Elisabeth Förster-Waldl, Kaan Boztug

Correspondence to: Kaan Boztug MD, CeMM Research Center for Molecular Medicine of the Austrian Academy of Sciences, Vienna & Ludwig Boltzmann Institute for Rare and Undiagnosed Diseases, Lazarettgasse 14 AKH BT 25.3, A-1090 Vienna;
e-mail: kboztug@cemm.oeaw.ac.at/ kaan.boztug@rud.lbg.ac.at; telephone number: +43 1 40160 70069; fax number: +43 1 40160 970000.

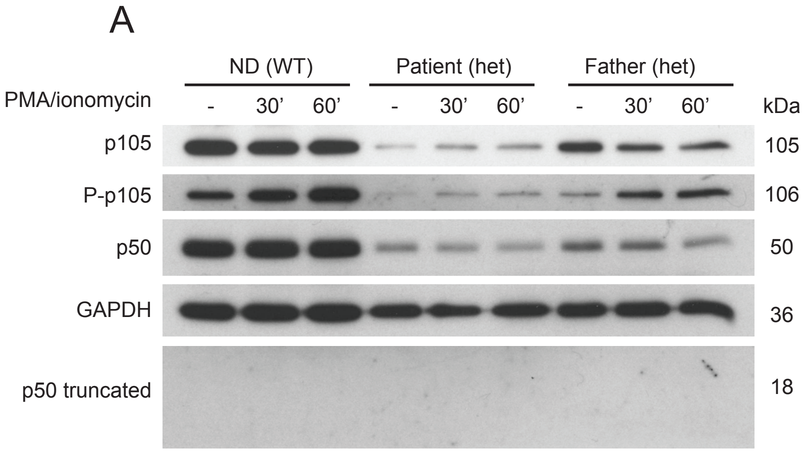

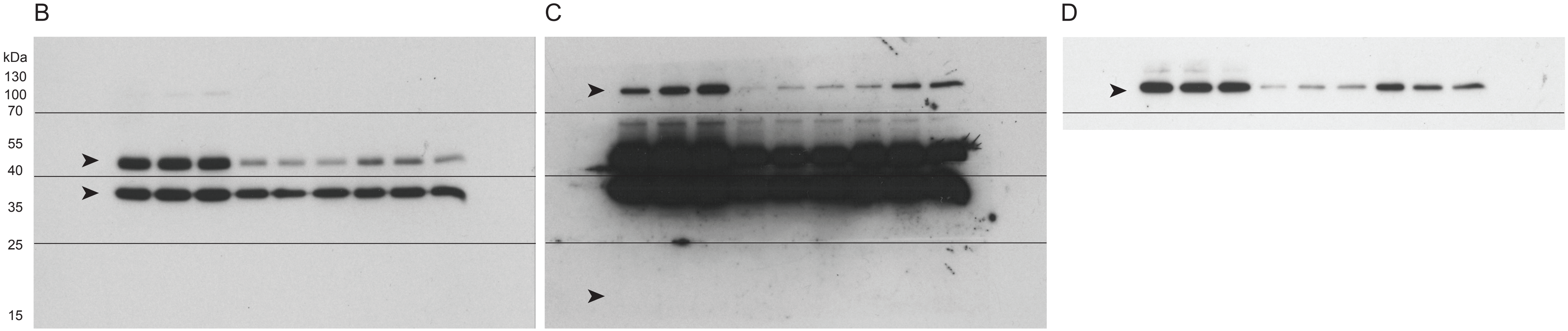

Supplementary Figure 1. Immunoblot analysis of index patient and her father.

A) Western blot as shown in Figure 2D with indicated molecular weight (kDa) of stained proteins.

B-D show original western blot films following different exposures. Horizontal lines indicate the cutting sites of membranes for the detection of multiple proteins on the same blot. The membrane was cut on the basis of PageRuler Prestained Protein Ladder (Thermo Scientific) marking points, as indicated on the left side. B) Top arrowhead indicates detected p50 protein; bottom arrowhead indicates detected GAPDH protein following 1 second of film exposure. C) Top arrowhead indicates detected P-p105 protein; bottom arrowhead indicates height of potentially expected truncated p50 protein following 10 seconds of film exposure. Due to lack of signal detection, proteasomal degradation of the non-full length protein expressed from the mutated allele can be assumed. D) Arrowhead indicates detected p105 protein following 10 seconds of film exposure. The staining was performed followng membrane stripping and is shown for completeness.
