# Supplementary material for: NF-κB1 Haploinsufficiency Causing Immunodeficiency and EBV-Driven Lymphoproliferation
Source: J Clin Immunol. 2016 Jun 23;36:533–40. doi: 10.1007/s10875-016-0306-1 (PMC4940442; doi:10.1007/s10875-016-0306-1)
Supplement: Supplementary file 2 — (DOCX 18 kb) [file 10875_2016_306_MOESM2_ESM.docx]

NF-κB1 haploinsufficiency causing combined immunodeficiency and EBV-driven lymphoproliferation

Journal of Clinical Immunology

Heidrun Boztug, Tatjana Hirschmugl, Wolfgang Holter, Karoly Lakatos, Leo Kager, Winfried Pickl, Elisabeth Förster-Waldl, Kaan Boztug

Correspondence to: Kaan Boztug MD, CeMM Research Center for Molecular Medicine of the Austrian Academy of Sciences, Vienna & Ludwig Boltzmann Institute for Rare and Undiagnosed Diseases, Lazarettgasse 14 AKH BT 25.3, A-1090 Vienna; e-mail: kboztug@cemm.oeaw.ac.at/kaan.boztug@rud.lbg.ac.at; telephone number: +43 1 40160 70069; fax number: +43 1 40160 970000.

**Supplementary Table 1. List of unique heterzygous variants detected in the index patient using a custom designed, NGS-based panel sequencing approach as described in Material and Methods.** No heterozygous variants were detected in the patient. Abbreviations: Chr, chromosome; Pos, position; ID, indication of rs number if applicable; ref, reference read; alt, alternative nucleotide in index patient; type, type of amino acid change; aa change, resulting amino acid change; transcript, transcript identifier; maf, minimal allele frequency; read, number of reads at the indicated position.

| **GENE** | **CHR** | **POS** | **ID** | **REF** | **ALT** | **TYPE** | **AA change** | **TRANSCRIPT** | **MAF** | **READS** |
| --- | --- | --- | --- | --- | --- | --- | --- | --- | --- | --- |
| *C4BPA* | 1 | 207317172 | rs151108669 | G | A | MISSENSE | R485Q | ENST00000367070 | < 0.01 | 241 |
| *C9* | 5 | 39341348 | rs199939436 | C | T | MISSENSE | G126R | ENST00000263408 | NA | 251 |
| *CFH* | 1 | 196709833 | rs145975787 | C | T | MISSENSE | T956M | ENST00000367429 | < 0.01 | 325 |
| *DSP* | 6 | 7559557 | rs377507763 | G | T | MISSENSE | C174F | ENST00000379802 | NA | 341 |
| *FASTKD1* | 2 | 170387132 | . | G | GT | FRAME SHIFT | p.R760Q*16 | ENST00000453929 | NA | 161 |
| *IL10RA* | 11 | 117869499 | . | C | T | MISSENSE | H145Y | ENST00000545409 | NA | 272 |
| *KMT2D* | 12 | 49445865 | . | G | A | MISSENSE | T534M | ENST00000301067 | NA | 93 |
| *NFKB1* | 4 | 103498115 | . | AG | A | FRAME SHIFT | p.G165A*31 | ENST00000394820 | NA | 107 |
| *VPS13B* | 8 | 100791164 | . | C | A | MISSENSE | L2562M | ENST00000357162 | NA | 15 |
