# Supplementary material for: NF-κB1 Haploinsufficiency Causing Immunodeficiency and EBV-Driven Lymphoproliferation
Source: J Clin Immunol. 2016 Jun 23;36:533–40. doi: 10.1007/s10875-016-0306-1 (PMC4940442; doi:10.1007/s10875-016-0306-1)
Supplement: Supplementary file 3 — (DOCX 31 kb) [file 10875_2016_306_MOESM3_ESM.docx]

**NF-κB1haploinsufficiency causing combined immunodeficiency and EBV-driven lymphoproliferation**

Journal of Clinical Immunology

Heidrun Boztug, Tatjana Hirschmugl, Wolfgang Holter, Karoly Lakatos, Leo Kager, Doris Trapin, Winfried Pickl, Elisabeth Förster-Waldl, Kaan Boztug

Correspondence to: Kaan Boztug MD, CeMM Research Center for Molecular Medicine of the Austrian Academy of Sciences, Vienna & Ludwig Boltzmann Institute for Rare and Undiagnosed Diseases, Lazarettgasse 14 AKH BT 25.3, A-1090 Vienna; e-mail: kboztug@cemm.oeaw.ac.at/kaan.boztug@rud.lbg.ac.at; telephone number: +43 1 40160 70069; fax number: +43 1 40160 970000.

**Supplementary Table 2. Coverage results for genes associated with EBV-lymphoproliferation such as *STK4, CD27, ITK*, *XIAP and SH2D1A,* using a custom designed NGS-based panel sequencing approach as described in Material and Methods.** Abbreviations: Chr, chromosome; Pos, position, MIN, minimun; MAX, maximum; UTR, untranslated region.

| ***STK4*** |  |  |  | **COVERAGE** | | |  |
| --- | --- | --- | --- | --- | --- | --- | --- |
| ENST00000372806 | | |  | (reads per exonic intervals) | | |  |
| **Exon** | **Chr** | **Pos1** | **Pos2** | **MIN** | **MEAN** | **MAX** | **COMMENT** |
| Exon1 | 20 | 43595115 | 43595244 | 111 | 186 | 306 |  |
| Exon2 | 20 | 43600719 | 43600799 | 25 | 131 | 196 |  |
| Exon3 | 20 | 43607084 | 43607212 | 151 | 165 | 206 |  |
| Exon4 | 20 | 43610470 | 43610584 | 151 | 190 | 195 |  |
| Exon5 | 20 | 43615773 | 43615937 | 19 | 131 | 229 |  |
| Exon6 | 20 | 43623731 | 43623898 | 119 | 564 | 638 |  |
| Exon7 | 20 | 43625810 | 43625947 | 123 | 290 | 446 |  |
| Exon8 | 20 | 43629033 | 43629161 | 309 | 366 | 467 |  |
| Exon9 | 20 | 43629808 | 43629994 | 48 | 257 | 352 |  |
| Exon10 | 20 | 43653614 | 43653771 | 50 | 219 | 399 |  |
| Exon11 | 20 | 43703659 | 43708600 | 0 | 208 | 880 | in 3'UTR |
|  |  |  |  |  |  |  |  |
| ***CD27*** |  |  |  | **COVERAGE** | | |  |
| ENST00000266557 | | |  | (reads per exonic intervals) | | |  |
| **Exon** | **Chr** | **Pos1** | **Pos2** | **MIN** | **MEAN** | **MAX** | **COMMENT** |
| Exon1 | 12 | 6554033 | 6554397 | 5 | 211 | 962 | in 5'UTR |
| Exon2 | 12 | 6554590 | 6554721 | 157 | 830 | 1015 |  |
| Exon3 | 12 | 6559339 | 6559518 | 261 | 483 | 1117 |  |
| Exon4 | 12 | 6559707 | 6559796 | 166 | 279 | 514 |  |
| Exon5 | 12 | 6560061 | 6560180 | 320 | 525 | 666 |  |
| Exon6 | 12 | 6560434 | 6560884 | 139 | 561 | 1228 |  |
|  |  |  |  |  |  |  |  |
| ***ITK*** |  |  |  | **COVERAGE** | | |  |
| ENST00000422843 | | |  | (reads per exonic intervals) | | |  |
| **Exon** | **Chr** | **Pos1** | **Pos2** | **MIN** | **MEAN** | **MAX** | **COMMENT** |
| Exon1 | 5 | 156607837 | 156608126 | 131 | 270 | 390 |  |
| Exon2 | 5 | 156635900 | 156636004 | 256 | 336 | 572 |  |
| Exon3 | 5 | 156638298 | 156638379 | 178 | 635 | 649 |  |
| Exon4 | 5 | 156641202 | 156641330 | 80 | 186 | 487 |  |
| Exon5 | 5 | 156644877 | 156644917 | 762 | 829 | 960 |  |
| Exon6 | 5 | 156649873 | 156650024 | 125 | 331 | 503 |  |
| Exon7 | 5 | 156655306 | 156655371 | 32 | 75 | 142 |  |
| Exon8 | 5 | 156659350 | 156659404 | 161 | 162 | 212 |  |
| Exon9 | 5 | 156665119 | 156665201 | 213 | 215 | 216 |  |
| Exon10 | 5 | 156667072 | 156667205 | 169 | 335 | 428 |  |
| Exon11 | 5 | 156668656 | 156668730 | 226 | 264 | 289 |  |
| Exon12 | 5 | 156670633 | 156670804 | 68 | 228 | 565 |  |
| Exon13 | 5 | 156671272 | 156671488 | 153 | 377 | 796 |  |
| Exon14 | 5 | 156672736 | 156672800 | 353 | 375 | 543 |  |
| Exon15 | 5 | 156672891 | 156673009 | 579 | 725 | 859 |  |
| Exon16 | 5 | 156675860 | 156676017 | 390 | 511 | 577 |  |
| Exon17 | 5 | 156679617 | 156682201 | 6 | 253 | 802 | in 3'UTR |
|  |  |  |  |  |  |  |  |
| ***XIAP*** |  |  |  | **COVERAGE** | | |  |
| ENST00000371199 | | |  | (reads per exonic intervals) | | |  |
| **Exon** | **Chr** | **Pos1** | **Pos2** | **MIN** | **MEAN** | **MAX** | **COMMENT** |
| Exon1 | X | 122993877 | 122994143 | 106 | 278 | 455 |  |
| Exon2 | X | 123019481 | 123020389 | 78 | 278 | 601 |  |
| Exon3 | X | 123022469 | 123022568 | 83 | 92 | 157 |  |
| Exon4 | X | 123025088 | 123025166 | 40 | 115 | 200 |  |
| Exon5 | X | 123026581 | 123026623 | 29 | 41 | 66 |  |
| Exon6 | X | 123034343 | 123034543 | 18 | 138 | 303 |  |
| Exon7 | X | 123040838 | 123047829 | 0 | 74 | 503 | in 3'UTR |
|  | | | | | | | |
| ***SH2D1A*** | | | | **COVERAGE** | | |  |
| ENST00000371139 | | | | (reads per exonic intervals) | | |  |
| **Exon** | **Chr** | **Pos1** | **Pos2** | **MIN** | **MEAN** | **MAX** | **COMMENT** |
| Exon1 | X | 123480194 | 123480629 | 139 | 502 | 805 |  |
| Exon2 | X | 123499611 | 123499674 | 78 | 146 | 315 |  |
| Exon3 | X | 123504026 | 123504170 | 243 | 442 | 576 |  |
| Exon4 | X | 123505201 | 123507005 | 0 | 144 | 664 | in 3'UTR |
